# The Effect of Neonatal Leptin Antagonism in Male Rat Offspring Is Dependent upon the Interaction between Prior Maternal Nutritional Status and Post-Weaning Diet

**DOI:** 10.1155/2012/296935

**Published:** 2012-04-02

**Authors:** J. Beltrand, D. M. Sloboda, K. L. Connor, M. Truong, M. H. Vickers

**Affiliations:** Liggins Institute and the National Research Centre for Growth and Development, University of Auckland, Auckland 1142, New Zealand

## Abstract

Epidemiological and experimental studies report associations between overweight mothers and increased obesity risk in offspring. It is unclear whether neonatal leptin regulation mediates this association between overweight mothers and offspring obesity. We investigated the effect of neonatal treatment with a leptin antagonist (LA) on growth and metabolism in offspring of mothers fed either a control or a high fat diet. Wistar rats were fed either a control (CON) or a high fat diet (MHF) during pregnancy and lactation. Male CON and MHF neonates received either saline (S) or a rat-specific pegylated LA on days 3, 5, and 7. Offspring were weaned onto either a control or a high fat (hf) diet. At day 100, body composition, blood glucose, **β**-hydroxybutyrate and plasma leptin and insulin were determined. In CON and MHF offspring, LA increased neonatal bodyweights compared to saline-treated offspring and was more pronounced in MHF offspring. In the post-weaning period, neonatal LA treatment decreased hf diet-induced weight gain but only in CON offspring. LA treatment induced changes in body length, fat mass, body temperature, and bone composition. Neonatal LA treatment can therefore exert effects on growth and metabolism in adulthood but is dependent upon interactions between maternal and post-weaning nutrition.

## 1. Introduction

Obesity and metabolic-related disorders are considered major health issues worldwide. Over the last decade, the incidence of obesity and overweight has almost doubled in developed countries and the trend is mirrored in developing nations that are transitioning to first-world economies [1]. Obesity results from an interaction of many factors including genetic, physiologic, behavioural, and environmental influences. However, the rapid increases in the rates of obesity suggest that environmental and behavioural influences, rather than genetic causes, are fuelling the present epidemic.

Increasing evidence from both clinical and animal studies has highlighted the link between altered maternal nutrition and the risk of offspring developing obesity and the metabolic syndrome [2–5]. Initial epidemiological studies suggested that fetal growth restriction is correlated with later disease, implying that fetal nutritional deprivation may be a strong stimulus for developmental programming. However, although maternal nutrient deprivation has been well characterized in this context, in many societies, maternal and postnatal nutrition can be excessive. As a result, excessive weight gain and/or obesity are common nutritional problems complicating pregnancy in developed countries. As such, there is now accumulating evidence from human [6] and animal studies suggesting that excess maternal caloric intake has adverse effects on the health and well-being of offspring, independent of postnatal diet [7] and exerted transgenerational effects [8].

Maternal obesity has many adverse outcomes, including labour and delivery complications, fetal and neonatal death, maternal hypertension, and preeclampsia and gestational diabetes [9–12]. In addition to acute risks to the obese mother, negative outcomes extend to offspring, including obesity and cardiovascular disease in adulthood [13–16]. On one hand, while a shared postnatal environment and genetic susceptibility are likely contributors [17], maternal BMI is reported in some cohorts as having greater influence on offspring than paternal BMI, highlighting the independent influence of the intrauterine environment on offspring adiposity [18].

In a rodent model, we recently reported that offspring born to high-fat-fed mothers showed an obese phenotype in adulthood characterised by hyperinsulinemia and hyperleptinemia, independent of postnatal diet [7, 19]. Although the mechanistic drivers underlying this obesogenic phenotype are still unclear, experimental data in rodents suggest that leptin plays a critical role in underpinning early life influences on postnatal phenotypic development [20–22]. Early studies demonstrated that leptin, an adipokine produced primarily by adipocytes, plays a key role in regulation of energy homeostasis and food intake via its action on specific hypothalamic nuclei [23]. Leptin has been since demonstrated to exert other important functions, including its regulation of bone growth, skeletal metabolism, and linear growth via direct effects on osteoblast and osteocalcin release and growth hormone secretion, respectively [24, 25].

In rodents, a characteristic of the neonatal period is a leptin surge, which normally peaks in the second week of neonatal life [26]. It has been shown that early life nutritional insults affect this surge resulting in altered hypothalamic development [27, 28]. Maternal undernutrition in rats has been shown to result in a blunted and altered timing of the leptin surge in neonatal pups [27], and leptin administration during the neonatal period in *ob*/*ob* mice normalised hypothalamic development and partially normalised orexigenic behaviour [21]. Importantly, postweaning leptin administration had no effect [27], emphasising that, in the rat, the critical stage of hypothalamic leptin regulation is during the first 2 weeks of neonatal life. Our group has previously shown that neonatal leptin treatment can reverse the deleterious effects of maternal undernutrition on postnatal outcomes in male and female offspring [29, 30]. In contrast, Kirk et al. reported that maternal high-fat diet led to an amplified leptin surge in neonatal pups during the first 2 weeks of life, resulting in altered hypothalamic regulation of food intake [31].

There is growing evidence that leptin plays a significant role in the development of an obesogenic phenotype after early-life exposure to an imprudent diet. Despite this, no studies have investigated whether maternal high-fat-diet-induced changes in neonatal leptin action regulate this association. We, therefore, hypothesized that leptin blockade, using a specific leptin antagonist, during the critical neonatal period of leptin sensitivity would ameliorate maternal high-fat-induced obesogenic effects on offspring [7]. We investigated the effect of leptin antagonist administration during the time of the critical neonatal leptin surge on weight gain, food intake, and body composition in male offspring born to mothers fed either a control or a high-fat diet.

## 2. Methods

### 2.1. Animal Model

**Figure 1:**
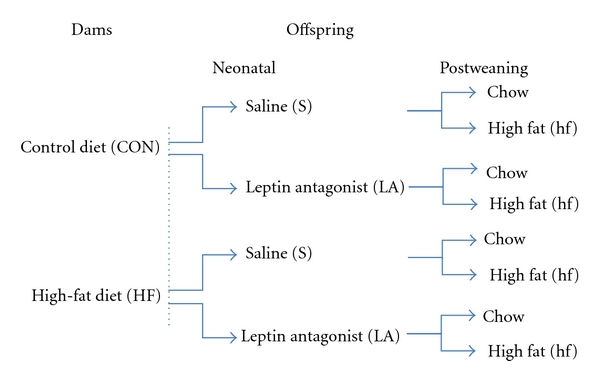
Schematic showing experimental design. There are two levels of maternal nutrition, 2 levels of neonatal treatment, and 2 levels of postweaning diet resulting in a total of 8 experimental groups in a fully balanced 2 × 2 × 2 design.

The animal model of maternal high-fat nutrition has been described in detail previously [7, 19]. Briefly, female Wistar rats were time-mated using a rat estrus cycle monitor (EC40, Fine Science Tools, Foster City, CA, USA) to assess the stage of estrus of the animal before introducing the male. Upon confirmation of mating, rats were randomly assigned to one of two maternal diets: the control chow diet throughout pregnancy and lactation (CON, *n* = 11 litters, Diet 2018, Harlan-Teklad, Oxon, UK) or a high-fat diet (MHF, *n* = 13 litters, 45% kcals from fat, D12451, Research Diets, New Brunswick, NJ, USA) to be fed *ad libitum* throughout pregnancy and lactation. Females were housed individually, with free access to water, and bodyweight and food intakes were measured every two days until the end of lactation. At birth, pups were weighed, and on postnatal day 2, litter size was adjusted to 8 pups per litter to ensure adequate and standardized nutrition until weaning. At postnatal day 3, MHF and CON litters were randomly assigned to receive either saline (S) or pegylated rat leptin antagonist (LA, mutant L39A/D40A/F41A, Protein Laboratories Rehovot, Israel). The LA or saline was administrated by subcutaneous injection at postnatal days 3, 5, and 7 at a dose of 12.5 *μ*g/g. Dosage and timing of LA administration was derived from calculated half-life (approximately 20 hours) and prior cited publications [32]. Male pups were weighed daily during the treatment period and then every 2 days thereafter until weaning (P22). At weaning, saline- and LA-treated CON and MHF male offspring were housed two per cage, and randomly placed on either the control rat chow (c) or high-fat (hf) diet until the completion of the trial (day 110). A schematic of the study design is shown in Figure 1.

Body weights and caloric intakes were recorded in offspring every 3 days until the end of the study. Body composition (fat mass, bone mineral content (BMC), and bone mineral density (BMD)) was measured by dual-energy X-ray absorptiometry (DEXA) at P100 under light isoflurane (2%) anaesthesia and using a dedicated small animal software package (Lunar Hologic, Waltham, MA). Rats were culled at P110 by decapitation following anaesthesia with sodium pentobarbitone (60 mg/kg). A tail blood sample was taken for fasting glucose and *β*-hydroxybutyrate (BHB) measurements (Roche Accucheck) and a rectal temperature measurement recorded. Trunk blood was collected into heparinised vacutainers, centrifuged and plasma stored at −20°C for later analysis. All animal work was approved by the Animal Ethics Committee of the University of Auckland.

### 2.2. Plasma Analyses

Plasma leptin and insulin concentrations were analysed using commercially available rat-specific ELISAs (no. 900040 and no. 90060, resp., CrystalChem, IL, USA).

### 2.3. Statistical Analysis

Data were analysed using JMP 7 (SAS Institute Inc., Cary, NC, USA) and R software (v.2.9.0, R Foundation for Statistical Computing, Vienna, Austria) for Windows. All data are presented as mean ± SEM unless otherwise stated. All models were statistically validated for assumptions of normality of residuals and absence of heteroscedasticity. Nonnormal data were log transformed to normalize where necessary. Maternal pregnancy data and neonatal data at birth were analysed using one-way ANOVA. Maternal caloric intakes; weight and body composition during lactation was analysed by two-way ANOVA with maternal diet and treatment group as factors and litter as covariate. Neonatal and postweaning growth was analysed by repeated measures factorial ANOVA. Although the growth analysis was performed on absolute body weight data, growth figures are shown as relative changes for sake of clarity given the number of experimental groups involved. Preweaning data for pups was analysed by two-way factorial ANOVA with maternal diet and LA administration as factors, and their interactions. Data from adult offspring were analyzed by three-way factorial ANOVA with maternal diet, postweaning diet, and LA treatment as factors, and the interaction between these factors (litter included as a covariate). Post hoc multiple pairwise comparisons were performed using Tukey test. Level of significance was set at *P* < 0.05.

## 3. Results

### 3.1. Maternal Data

**Figure 2:**
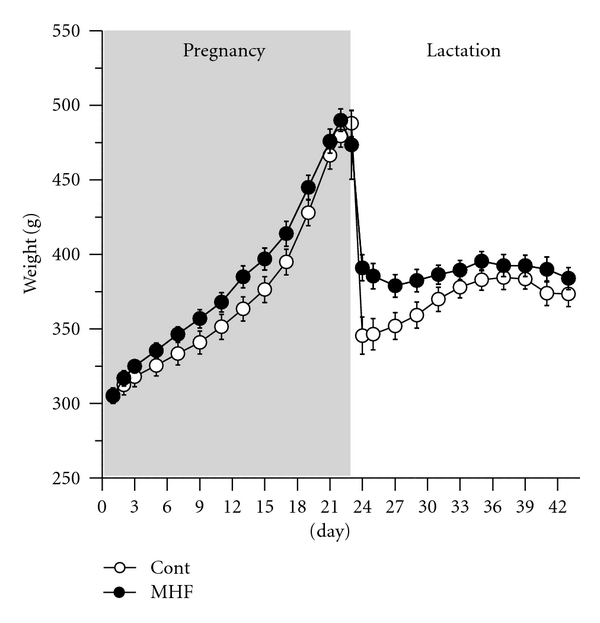
Maternal body weights during pregnancy and lactation. There was no overall significant effect of neonatal LA administration on weight of dams during pregnancy and lactation thus data for Sal and LA groups for each respective maternal diet have been combined. Data are means ± SEM with a minimum of 5 litters per group.

Consistent with our previous observations [7], maternal HF diet during pregnancy and lactation resulted in a transient increase in caloric intake from day 2 (*P* < 0.001) to day 15 of gestation when intakes returned to levels similar to those observed in CON dams (data not shown). Increased caloric intake in MHF dams was reflected in an increased maternal weight gain by gestational day 7 (*P* = 0.04, Figure 2), which persisted until birth. MHF dams remained heavier than controls from the early neonatal period until mid-lactation when body weights returned to match those of controls. There was no overall significant effect of neonatal LA administration on weight or caloric intake of dams during pregnancy and lactation. At weaning, despite a similar maternal body weight, total fat mass (%) and fat : lean ratios (F/L) were significantly increased in MHF dams compared to CON (% fat: CON 14.7 ± 1.7% versus MHF 22.9 ± 2.3, *P* = 0.01; F/L: CON 0.18 ± 0.08 versus MHF 0.31 ± 0.16, *P* = 0.01). There was no significant effect of neonatal LA treatment or an interaction between treatment and maternal diet, on body composition of dams.

### 3.2. Neonatal Growth

Birthweights were slightly but significantly reduced in male offspring of MHF dams compared to CON (CON 6.2 ± 0.1 g; MHF 5.9 ± 0.1 g, *P* < 0.001). At P3 and prior to start of LA administration, pups born to MHF mothers remained lighter than CON neonates (CON 7.1 ± 0.1 g; MHF 6.8 ± 0.1 g, *P* < 0.05).

**Figure 3:**
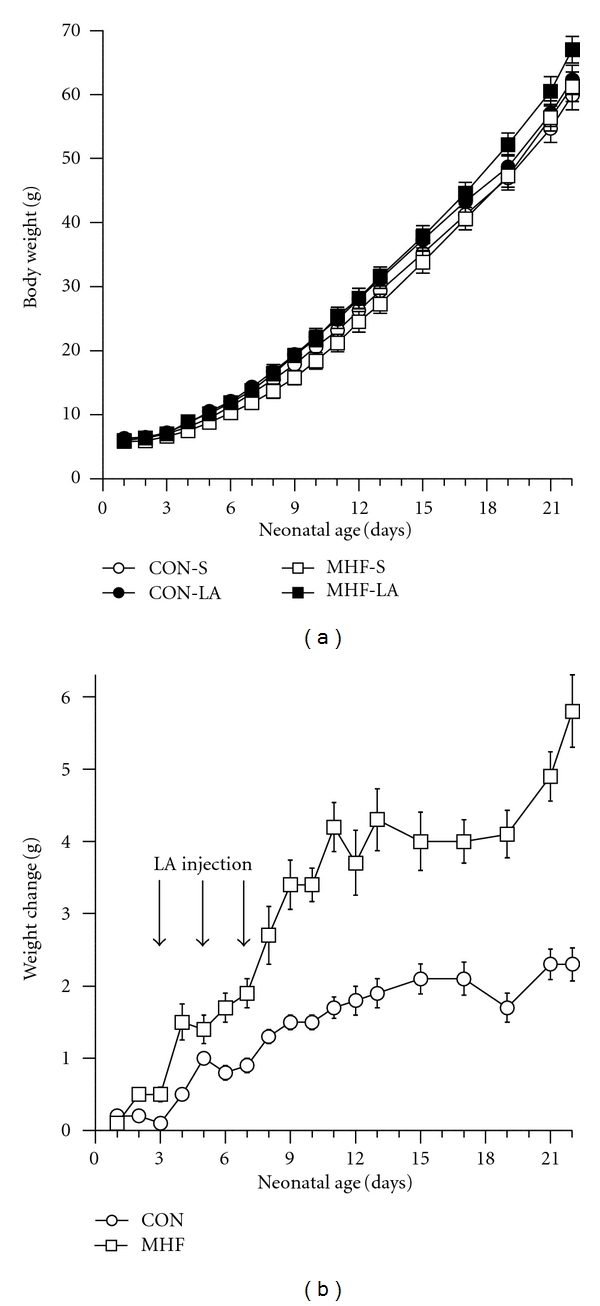
(a) Absolute body weights in male offspring from birth until weaning and (b) changes in neonatal body weights (grams, shown as delta difference in body weight between LA and Sal treated offspring, ΔLA-Sal) in CON and MHF neonates from birth until the time of weaning (day 22). Arrows indicate time of injection with LA at neonatal days 3, 5, and 7. *P* < 0.005 for effect of LA treatment; maternal diet × LA treatment interaction *P* < 0.05. *N* = minimum 16 per group.

LA administration leads to an increased neonatal weight gain in CON and MHF offspring compared to their saline treated counterparts (*P* < 0.005, Figure 3). The increased weight gain in LA-treated neonates was more pronounced in offspring of MHF dams reflected in a maternal diet × LA treatment interaction (*P* < 0.005). By weaning (P22), MHF offspring were slightly but significantly heavier than CON offspring and LA treatment further increased weaning weights in CONT and MHF offspring (P22: CON-S 59.9 ± 0.9 g, CON-LA 62.0 ± 1.1 g, MHF-S 61.8 ± 1.7 g, MHF-LA 66.3 ± 1.1 g, *P* < 0.05 for effect of maternal diet and LA treatment, no interactions).

### 3.3. Postweaning Growth and Body Composition

**Figure 4:**
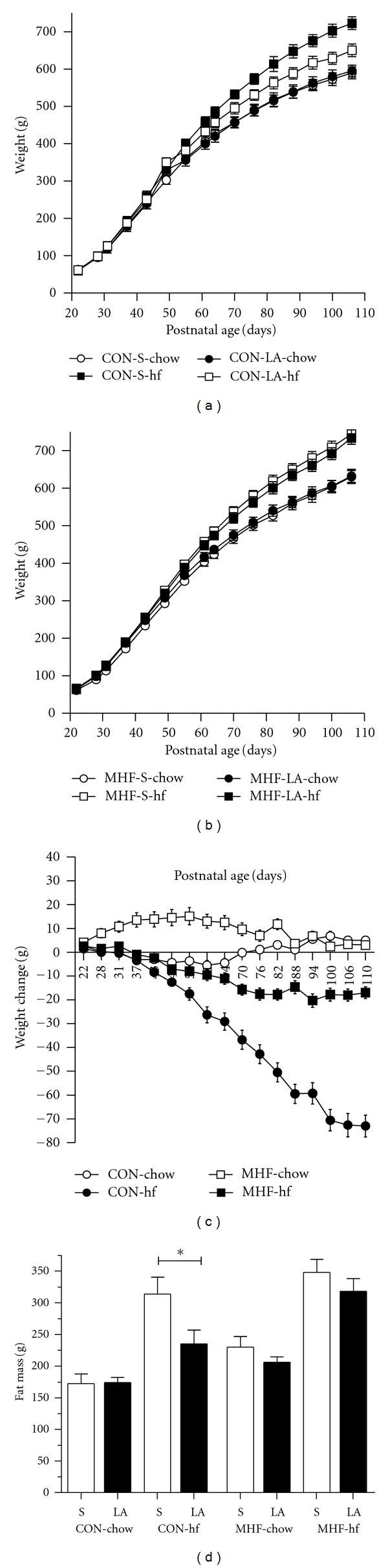
Body weights in male offspring of either CON (a) or MHF (b) mothers treated with either saline or LA as neonates and fed either a chow or hf diet after weaning. Data are means ± SEM, *n* = minimum 8 per group; (c) change in body weight (grams, shown as delta difference in body weight between LA and Sal-treated offspring, ΔLA-Sal) in CON and MHF offspring fed either a control chow or hf diet from weaning until postnatal day 110. Data are means ± SEM, *n* = minimum 8 per group. Maternal diet × LA treatment interaction *P* < 0.005; (d) fat mass (g) as quantified by DEXA scanning in offspring of CON of MHF dams, treated with either Sal or LA as neonates and fed either a control chow or hf diet after weaning. Data are means ± SEM, minimum of 8 per group. *P < 0.05.

An MHF diet had no significant effect on adult body weight at postnatal day 110 but resulted in significantly increased total percent body fat and decreased lean body weight percentage compared to CON animals as quantified by DEXA scanning (Table 1). A postweaning hf diet increased body weight and total body fat mass in all hf-fed groups. Neonatal LA treatment had a significant overall effect on reducing total percent body fat mass, increasing lean mass and a decreased fat : lean ratio (Table 1). A significant maternal diet × LA treatment × postnatal diet interaction (*P* < 0.001) revealed that body weights were significantly reduced in offspring of CON dams that were treated as neonates with LA and fed a postweaning hf diet as compared to saline treated CON offspring fed the hf diet (Figure 4(c)). LA treatment in CON neonates reduced hf diet-induced obesity by approximately 10% and equated to an absolute bodyweight difference of 72.6 g (Figure 4(a)). DEXA analysis of body fat content showed that this reduction in body fat was paralleled by a reduction in fat mass in these animals compared to saline treated (Figure 4(d)). This effect was not observed in MHF offspring where neonatal LA failed to significantly impact on postweaning hf-induced changes in final bodyweight or fat mass (Figures 4(b) and 4(d) and Table 1). This may reflect a more marked increase in relative lean mass in LA-treated CON-hf offspring as compared to LA treated MHF-hf offspring compared to relative saline-treated groups (Table 1).

**Figure 5:**
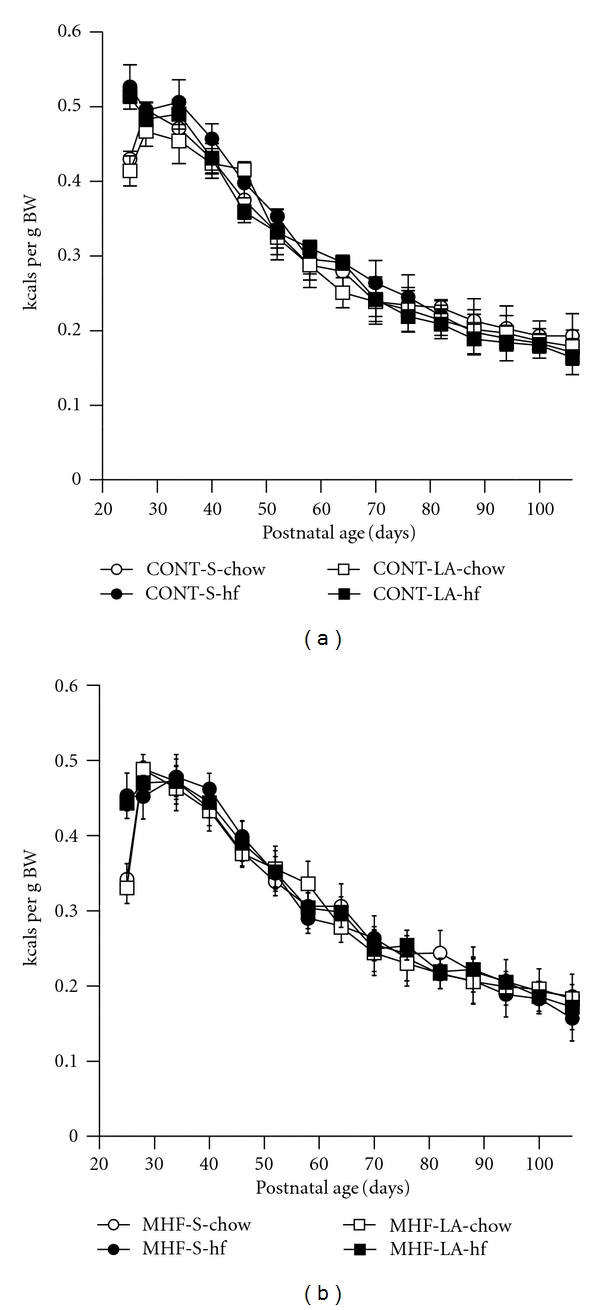
Total caloric intake (kcals consumed per gram body weight) in male offspring of CON mothers (a) or MHF mothers (b) from birth until the completion of the trial. Data are average caloric intakes per cage of 2 animals, minimum of 4 cages per group.

There were no significant effects of MHF diet, neonatal LA treatment, or postweaning hf diet on total caloric intake (expressed as kcals consumed per gram body weight) across any of the treatment groups (Figures 5(a) and 5(b)).

There were significant overall effects of maternal diet and LA treatment on nose-anus (NA) length (Table 1). Post hoc analysis revealed LA treatment increased NA length in MHF offspring but not CON offspring as reflected in a maternal diet × LA treatment interaction (*P* < 0.05). A postweaning hf diet increased NA length only in MHF offspring (Table 1).

Maternal diet had no overall effect on nose-tail (NT) length (Table 1). There were overall significant effects of neonatal LA treatment and postweaning hf diet on increasing NT length. A significant maternal diet × LA treatment interaction revealed that increases in NT length as a result of neonatal LA treatment were greater in MHF offspring compared to CON offspring for both chow and postweaning hf diets (Table 1).

### 3.4. Bone Mineral Density (BMD)

There was no effect of MHF diet on BMD (Table 1). Neonatal LA treatment reduced BMD in all treatment groups and a postweaning hf diet increased BMD in all offspring. There were no statistically significant interactions.

### 3.5. Bone Mineral Content (BMC)

An MHF diet had the overall effect of increasing BMC in all offspring (Table 1). Neonatal LA treatment reduced BMC in all treated groups and a postweaning hf diet increased BMC in all offspring. There were no statistically significant interactions.

### 3.6. Rectal Temperature (RT)

An MHF diet significantly increased RT in all MHF offspring (Table 2). Neonatal LA treatment reduced RT in all treatment groups. A postweaning hf diet had no significant effect on RT (*P* = 0.092). There were no statistically significant interactions.

### 3.7. Leptin, Insulin, Glucose, and *β*-Hydroxybutyrate (BHB) Levels

An MHF diet significantly increased plasma leptin levels in all MHF offspring (Table 2). Neonatal LA treatment had no overall effect on plasma leptin levels. A postweaning hf diet increases plasma leptin in all hf-fed offspring. There were no statistically significant interactions for plasma leptin. There was a strong trend toward increased fasting plasma insulin levels in MHF offspring, but this difference did not reach statistical significance (*P* = 0.068). LA treatment had no effect on plasma insulin levels. A postweaning hf diet increased plasma insulin levels in all hf-fed groups (Table 2). Fasting plasma glucose levels were similar between the groups (Table 2). Blood BHB levels were not altered by MHF diet or neonatal LA treatment but were significantly increased in all hf-fed offspring compared to chow-fed offspring (Table 2).

## 4. Discussion

These results have demonstrated for the first time that early-life manipulation of the leptin axis via neonatal leptin antagonism can exert marked effects on growth and body composition, which are dependent upon prior maternal nutrition status and postweaning diet. Investigators using neonatal leptin treatment given to offspring of normally fed dams have shown increased adiposity and leptin and insulin resistance in offspring in later life [33–35]. The present result shows that the reverse can hold true. Control offspring, given a leptin antagonist prior to being fed an obesogenic hf diet postweaning, show an amelioration of a diet-induced fat accumulation and reduced linear body growth. The marked contrast in adult phenotype in offspring of normally nourished mothers, based on exposure to either leptin or leptin antagonism during early-life development, further serves to highlight how critical the maintenance of leptin threshold levels is during this period of developmental plasticity.

As we have shown previously [7], maternal high-fat nutrition resulted in increased adiposity, leptin, and insulin concentrations in offspring compared to offspring of control mothers, independent of postweaning diet. There is a well-characterized leptin surge in the first two weeks of life in the rodent [26] although the source of the leptin is yet to be defined with the surge occurring independently of changes in neonatal body weight trajectory and milk leptin intake [31]. We have previously shown that offspring of MHF mothers are hypoleptinemic at birth [7]. This concurs with the increased sensitivity to body weight gain in MHF neonates treated with the LA as compared to CON offspring. In other studies, hypoleptinemic offspring of mothers undernourished during pregnancy have either a delayed [36] or premature leptin surge [27]. However, there is little known about the leptin surge in models of maternal obesity. Kirk et al. recently reported that rat offspring of mothers fed an obesogenic diet had normal serum leptin levels at birth but displayed an amplified and prolonged neonatal leptin surge, which was accompanied by an elevation in leptin mRNA expression in abdominal white adipose tissue [31]. However, it is unknown whether the leptin surge in the MHF offspring of the present study is altered.

Although inborn leptin deficiency causes weight gain, it is unclear whether induced leptin deficiency in adult wild-type animals would be orexigenic. Leptin antagonists have only recently become commercially available and provide an invaluable tool for investigating central and peripheral leptin deficiency and exploring the involvement of leptin in metabolic processes. Previous reports using a nonpegylated leptin antagonist have been problematic. The extremely short half-life of the antagonist necessitated administration of supraphysiological doses to induce a clinical response and was not sufficient to induce a true metabolic state of leptin deficiency [32, 37]. Hormones with molecular masses similar to that of leptin are cleared primarily via the kidney with a half life of only 8–30 minutes [38].

The effect of early postnatal leptin blockade in normal rat neonates has previously been reported in the study by Attig et al. [39]. In this work, the authors studied the long-term effect of neonatal therapy with a non-pegylated leptin antagonist (day 2 to day 13) in female Wistar rats [39]. In contrast to the present study, they showed that leptin antagonism induced a decrease in neonatal weight gain, which has previously been commonly associated with neonatal leptin treatment [30, 40]. Later in life, the leptin disruption led to a higher sensitivity to diet-induced obesity, as shown by a higher body weight gain when challenged with a high-energy diet, associated with increased adiposity and leptinemia. These animals also displayed a phenotype of leptin resistance at 4 months, characterized by the inability of treated animals to respond to leptin by failing to reduce food intake and showing reduced birth weight. Overall, the long-term effect in the Attig study was paradoxically similar to that reported for rats treated with leptin during neonatal life [30, 40]. Importantly, the molecule used as the leptin antagonist was different from the one used in our study. Indeed in this work, the authors used the leptin mutein, a molecule acting as an antagonist, with *in vivo* effects previously validated only using intracerebroventricular, but not subcutaneous, administration [41–44]. Furthermore, this antagonist, obtained by alanine mutagenesis of amino acids 39 to 41-42, has an extremely short half-life and high doses are required to produce a clinical response that is similar to a true metabolic state of leptin resistance.

The present study utilised a recently developed rat-specific pegylated LA whereby the attachment of polyethylene glycol increased the overall molecule size to 70 kDa. Pegylation of the LA results in an approximate 30-fold increase in *in vivo* half-life [32], thus true states of induced leptin deficiency are possible at physiologic doses. To date, only one prior study has examined the effects of the pegylated LA moiety, albeit in normal postweaning animals [37] where it was shown that treatment with the pegylated LA to postweaning mice results in a rapid and dramatic increase in food intake and weight gain [32]. The blood brain barrier (BBB) in the neonatal rat is relatively immature; pegylated leptin antagonist has been shown to block circulating leptin from crossing the BBB, an action that would attenuate the anorexigenic effect of leptin [32]. It is difficult to extrapolate the Elinav et al. study to the present study. The windows of treatment are different, BBB permeability is at different developmental stages (neonatal versus postweaning), and offspring responsiveness to leptin intervention is known to elicit sexually dimorphic responses [29, 30]. In addition, the work in the mouse examined the immediate phenotypic response to LA treatment, whereas the present study examines a postnatal phenotype derived from an early-life neonatal intervention. However, consistent with the reports from mice, the present result demonstrated that LA treatment induced a significant increase in body weight over the neonatal treatment period.

In the present study, neonatal leptin antagonism, despite having significant effects on pre-weaning weights in offspring of MHF mothers, had no effect on postnatal weight gain in CON or MHF offspring fed the standard chow diet. There was, however, a marked effect of neonatal LA treatment in reducing body weight gain in CON offspring fed the hf diet after weaning. Conversely, LA treatment to MHF offspring subsequently fed the hf diet had no significant effect on body weight; independent of changes in body weight and circulating plasma leptin concentrations. LA treatment significantly reduced fat deposit weight in CON but not MHF offspring. Interestingly, neonatal LA treatment did not alter postweaning caloric intake, thus the observed changes in body weight gain are independent of food intake and suggest a lack of effect of LA administration on the arcuate nucleus and related feeding circuitry, as has been reported with neonatal leptin treatment in the *ob/ob* mouse [21].

Nose-anus lengths were increased in MHF offspring but not CON offspring, which may suggest that altered effects on the growth-hormone- (GH-) insulin-like growth factor (IGF) axis are mediated by neonatal LA exposure. The observed change in tail length in CON and MHF LA-treated offspring was unexpected but may have resulted from altered thermoregulatory set-point processes as reflected in the significant differences in basal body temperature. In the rat, a significant portion of total body heat loss occurs through sympathetically mediated changes in tail blood flow [45]. However, since rectal temperature was decreased in LA-treated CON *and* MHF offspring, it is difficult to explain the disparate changes in tail length to thermoregulatory processes and, as with nose-anus length, may reflect LA-induced alterations in the GH-IGF axis in MHF offspring as compared to controls or development of a thrifty metabolic phenotype as regards thermogenesis and energy expenditure. Future independent studies looking at brown fat thermogenesis and uncoupling proteins may further explain this observation.

The effect of LA on bone formation has not previously been described. Bone morphology was significantly altered in adult offspring following neonatal LA treatment with overall significant reductions in BMC and BMD. It is well established that leptin treatment can result in enhanced bone formation and promotion of pro-osteogenic factors in bone marrow [46, 47], and the current data suggests that the reverse holds true for leptin antagonism and further work investigating specific bone markers is now warranted.

This is the first study designed to examine the efficacy of neonatal leptin antagonism following altered maternal nutrition and its interaction with differing levels of postweaning nutrition, on offspring phenotype development. Responsiveness to neonatal leptin antagonism is dependent upon both maternal and postweaning nutrition, with minimal efficacy in chow-fed offspring of either CON or MHF mothers. More studies are now required to further understand the mechanistic underpinnings of the present observations, including characterization of the effects of leptin antagonism on the timing and magnitude of the leptin surge in offspring of mothers with different dietary backgrounds. However, it is important to recognise that leptin-mediated development of feeding circuits occurs postnatally in the rodent and occurs *in utero* in primates, including humans, and thus timing of intervention strategies may be different. Nonetheless, taken together, the data on both neonatal leptin treatment and leptin antagonism in the setting of both normal and nutritionally challenged pregnancies serves to highlight the important role of leptin regulation during critical early-life windows of development on lasting growth and metabolic function in offspring.

## Figures and Tables

**Table 1:** Adult (P110) body weight, total fat (%), body length, bone density, and mineral content in CON or MHF offspring treated with either saline (S) or pegylated leptin antagonist (LA) as neonates and fed either a chow or hf diet after weaning. Data are means ± SEM, *n* = 8–16 per group.

Group	Final body weight (g)	Total body Fat (%)	Lean mass (%)	Fat : lean ratio	Nose-anus (mm)	Nose-tail (mm)	BMD (g/cm^2^)	BMC (g)
CON-S-chow	590 ± 10	31.0 ± 2.3	67.2 ± 2.2	0.47 ± 0.05	277 ± 2	478 ± 2	0.174 ± 0.001	14.8 ± 0.4
CON-S-hf	723 ± 19	46.6 ± 2.6	52.0 ± 2.5	0.91± 0.09	279 ± 2	484 ± 4	0.183 ± 0.003	17.6 ± 0.6
CON-LA-chow	595 ± 17	29.1 ± 1.1	69.3 ± 0.9	0.43 ± 0.02	277 ± 3	479 ± 4	0.171 ± 0.002	14.5 ± 0.2
CON-LA-hf	650 ± 21	37.9 ± 3.1	60.0 ± 3.8	0.72 ± 0.09	278 ± 2	489 ± 3	0.174 ± 0.003	15.8 ± 0.6
MHF-S-chow	621 ± 11	38.9 ± 2.1	59.6 ± 2.1	0.65 ± 0.06	277 ± 2	477 ± 3	0.176 ± 0.002	15.7 ± 0.3
MHF-S-hf	729 ± 18	51.0 ± 1.6	47.8 ± 1.5	1.06 ± 0.07	280 ± 2	480 ± 3	0.187 ± 0.003	18.7 ± 0.4
MHF-LA-chow	624 ± 13	34.9 ± 1.5	63.5 ± 1.5	0.54 ± 0.03	283 ± 1	490 ± 2	0.171 ± 0.001	14.8 ± 0.3
MHF-LA-hf	710 ± 18	44.1 ± 3.0	50.8 ± 1.9	0.94 ± 0.08	287 ± 2	493 ± 4	0.179 ± 0.002	17.5 ± 0.4

Main effects								
Maternal diet (MD)	*P* < 0.05	*P* < 0.005	*P* < 0.0001	*P* < 0.005	*P* < 0.05	NS	NS	*P* < 0.005
LA treatment (LA)	NS	*P* < 0.005	*P* < 0.05	*P* < 0.05	*P* < 0.05	*P* < 0.005	*P* < 0.0001	*P* < 0.005
PW-diet	*P* < 0.0001	*P* < 0.0001	*P* < 0.0001	*P* < 0.0001	NS	*P* < 0.05	*P* < 0.0001	*P* < 0.0001

Interactions								
MD × LA	NS	NS	NS	NS	*P* < 0.05	*P* < 0.05	NS	NS
LA × PW-diet	*P* = 0.05	NS	NS	NS	NS	NS	NS	NS
MD × PW diet	NS	NS	NS	NS	NS	NS	NS	NS
MD × PW diet × LA	NS	NS	NS	NS	NS	NS	NS	NS

**Table 2:** Fasting plasma leptin, insulin, glucose, and *β*-hydroxybutyrate (BHB) measurements and rectal temperature in CON and MHF offspring treated with either saline (S) or pegylated leptin antagonist (LA) as neonates and fed either a chow or hf diet after weaning. Data are means ± SEM, *n* = 8–16 per group, no significant statistical interactions.

	Leptin (ng/mL)	Insulin (ng/mL)	Glucose (mmol/L)	BHB (mmol/L)	Rectal Temperature (°C)
CON-S-chow	10.4 ± 0.8	2.9 ± 0.2	5.98 ± 0.26	1.07 ± 0.07	37.19 ± 0.18
CON-S-hf	34.1 ± 3.6	4.6 ± 0.4	6.06 ± 0.32	1.57 ± 0.14	37.14 ± 0.13
CON-LA-chow	11.2 ± 1.4	3.4 ± 0.6	6.07 ± 0.37	1.24 ± 0.14	36.63 ± 0.19
CON-LA-hf	24.4 ± 3.9	5.9 ± 0.7	5.91 ± 0.27	1.29 ± 0.16	37.08 ± 0.14
MHF-S-chow	20.9 ± 2.9	4.4 ± 0.9	5.96 ± 0.26	0.92 ± 0.08	37.32 ± 0.15
MHF-S-hf	36.1 ± 3.0	6.1 ± 0.7	5.82 ± 0.32	1.47 ± 0.10	37.63 ± 0.21
MHF-LA-chow	16.7 ± 2.8	4.0 ± 0.6	5.28 ± 0.34	1.05 ± 0.14	37.08 ± 0.19
MHF-LA-hf	31.8 ± 4.2	6.1 ± 0.9	5.24 ± 0.31	1.61 ± 0.14	37.28 ± 0.11

Main effects					
Maternal diet	*P* < 0.05	*P* < 0.05	NS	NS	*P* < 0.05
LA treatment	NS	NS	NS	NS	*P* < 0.05
PW-diet	*P* < 0.0001	*P* < 0.0001	NS	*P* < 0.0001	NS
